# A genetic selection for *Mycobacterium smegmatis* mutants tolerant to killing by sodium citrate defines a combined role for cation homeostasis and osmotic stress in cell death

**DOI:** 10.1128/msphere.00358-23

**Published:** 2023-09-08

**Authors:** John T. Williams, Jacob J. Baker, Huiqing Zheng, Shelby J. Dechow, Jared Fallon, Megan Murto, Veronica J. Albrecht, Haleigh N. Gilliland, Andrew J. Olive, Robert B. Abramovitch

**Affiliations:** 1 Department of Microbiology and Molecular Genetics, Michigan State University, East Lansing, Michigan, USA; The University of Iowa, Iowa City, Iowa, USA

**Keywords:** *Mycobacterium*, stress response, metals

## Abstract

Mycobacteria can colonize environments where the availability of metal ions is limited. Biological or inorganic chelators play an important role in limiting metal availability, and we developed a model to examine *Mycobacterium smegmatis* survival in the presence of the chelator sodium citrate. We observed that instead of restricting *M. smegmatis* growth, concentrated sodium citrate killed *M. smegmatis*. RNAseq analysis during sodium citrate treatment revealed transcriptional signatures of metal starvation and hyperosmotic stress. Notably, metal starvation and hyperosmotic stress, individually, do not kill *M. smegmatis* under these conditions. A forward genetic transposon selection was conducted to examine why sodium citrate was lethal, and several sodium-citrate-tolerant mutants were isolated. Based on the identity of three tolerant mutants, *mgtE*, *treZ*, and *fadD6,* we propose a dual stress model of killing by sodium citrate, where sodium citrate chelate metals from the cell envelope and then osmotic stress in combination with a weakened cell envelope causes cell lysis. This sodium citrate tolerance screen identified mutants in several other genes with no known function, with most conserved in the pathogen *M. tuberculosis*. Therefore, this model will serve as a basis to define their functions, potentially in maintaining cell wall integrity, cation homeostasis, or osmotolerance.

**IMPORTANCE:**

Bacteria require mechanisms to adapt to environments with differing metal availability. When *Mycobacterium smegmatis* is treated with high concentrations of the metal chelator sodium citrate, the bacteria are killed. To define the mechanisms underlying killing by sodium citrate, we conducted a genetic selection and observed tolerance to killing in mutants of the *mgtE* magnesium transporter. Further characterization studies support a model where killing by sodium citrate is driven by a weakened cell wall and osmotic stress, that in combination cause cell lysis.

## INTRODUCTION

Metal cations play crucial roles in physiologies required for bacterial viability, including protein structure and function, DNA/RNA polymerization (1), and cell envelope stability (2 – 9). To this end, bacteria include a variety of metal import and export systems to maintain stable cytoplasmic cation concentrations. The most abundant divalent cation present in bacteria is Mg^2+^ which plays a major role in cell envelope stability (2 – 9), and it is estimated that >30% of Mg^2+^ is stored in the cell wall of *Escherichia coli* (5). In both Gram-positive and Gram-negative bacteria, Mg^2+^ serves as one of the major counterions to offset negative charges of cell envelope components such as (wall) teichoic acids [(W)TA] (7 – 9), lipopolysaccharides (LPS) (3 – 5), and peptidoglycan (8, 9). Mycobacteria contain neither (W)TA nor LPS but do contain peptidoglycan (6) and phospholipids such as phosphatidylinositol mannosides (PIMs) that contain negatively charged phosphate residues (10). However, the Mg^2+^ content of the mycobacterial cell envelope is not well characterized and methods for measuring cation concentrations in live bacterial cells still remain elusive.

In addition to metal homeostasis, bacteria must also maintain osmotic homeostasis to maintain cell shape and integrity. In conditions with high environmental osmolytes, such as sodium, bacteria will respond using multiple strategies, including the import of environmental salts to counter osmotic pressures (11) or the accumulation of non-ionic compatible solutes, such as ectoine or betaine, in the cytoplasm (11, 12). Mycobacteria use the latter of the two strategies: either synthesize ectoine (13) or import host-derived betaine into the cytoplasm to offset osmotic pressures (14). Ectoine biosynthesis in the environmental model mycobacterium species *Mycobacterium smegmatis* allows for adaptation to high-saline environments (13). However, the genes required for this (*ectA-ectC* and *thpD*) are not conserved in all mycobacteria. The pathogenic species *M. tuberculosis* (Mtb) does not encode genes for ectoine biosynthesis (15) and relies on the import of host-derived betaine/glycine through the ABC transporter ProXVWZ (14) to adapt to host-induced osmotic pressure (16). This import system is regulated by the Ser/Thr kinase PknD and the anti-anti-sigma factor OprA (*Rv0516c*) which responds to high saline (17). Mycobacteria lacking these osmotic stress adaptation pathways have reduced growth (13, 14, 17) and pathogenicity in high saline and intracellular infection models (14, 17).

We previously reported on the metabolic adaptation of Mtb to acidic pH in a minimal medium supplemented with different carbon sources. In these studies, Mtb enters into the state of non-replicating persistence when cultured in an acidic medium supplemented with carbon sources such as glycerol but could grow in the presence of carbon sources that feed into the anaplerotic node (18, 19). To further study this system, we attempted to recapitulate this phenotype in the rapidly growing mycobacterium model organism *M. smegmatis*. However, early attempts at recreating this phenotype showed that *M. smegmatis* was less sensitive to acidic (pH 5.7) environments than Mtb. Therefore, we lowered the pH further using sodium citrate (100 mM) buffered medium. However, this induced a lethal phenotype in *M. smegmatis* that was more pronounced at neutral pH (pH 7.0) conditions and dependent on carbon supplementation. Transcriptional profiling indicated that *M. smegmatis* was responding to chelation and osmotic stress in a sodium citrate medium. Metal chelation or osmotic stress alone is generally not lethal to *M. smegmatis*; therefore, we hypothesized that a more complex mechanism underlay the killing of *M. smegmatis*. Given a strong phenotype for selection, we performed a forward genetic transposon mutagenesis selection for mutants that are not killed by sodium citrate treatment. The selection identified sodium-citrate-tolerant mutants through disruption of *mgtE*, *fadD6, treZ*, and several other seemingly unrelated genes. Based on characterization studies of these mutants, we propose a dual stress model in which sodium citrate results in the chelation of magnesium and calcium from the cell envelope. This cell wall metal chelation leads to cell wall weakening and in combination with osmotic stress causes cell lysis and death. We propose that this dual stress model of osmotic stress and weakened cell walls could serve as the basis to define the function of other mutants in genes that promote citrate tolerance but have no known function.

## MATERIALS AND METHODS

### Bacterial strains and growth conditions

Bacterial strains were cultured in the following conditions unless otherwise described. *M. smegmatis* MC2-155 was cultured in LB medium with 0.05% Tween-80 shaking at 120 rpm at 37°C in T25 or T75 flasks. Mtb was cultured in 10 mL of 7H9 supplemented with 10% (vol/vol) oleic albumin dextrose catalase (OADC) with 0.05% Tween-80 in T25 standing flasks. *M. abscessus* was cultured in LB with 0.05% Tween-80 shaking at 200 rpm at 37°C.

MMAT minimal medium was made as previously described (20): 1 g/L KH_2_PO_4_, 2.5  g/L Na_2_PO_4_, 0.5  g/L (NH_4_)_2_SO_4_, 0.15  g/L asparagine, 10 mg/L MgSO_4_, 50 mg/L ferric ammonium citrate, 0.1 mg/L ZnSO_4_, 0.5  mg/L CaCl_2_, and 0.05% Tyloxapol. MMAT medium was buffered with 100 mM MOPS (pH 7.0), MES (pH 5.7), and sodium citrate (pH 5.0–7.0). MMAT was supplemented with 10 mM glucose or 10 mM glycerol as carbon sources.

### Growth curves

*M. smegmatis* was grown to exponential growth (0.6–1.0) in LB with 0.05% Tween-80 at 37°C in T25 standing flasks. Samples were washed twice in phosphate buffer saline (PBS, pH 7.4) with 0.05% tyloxapol (PBS-T) and resuspended in MMAT medium buffered to pH 7.0 with 100 mM sodium citrate and supplemented with 10 mM glycerol at a starting optical density (OD)_600_ of 0.05. At the indicated time points, 1 mL of samples were taken and measured for cell growth (OD_600_). The limit of detection for this assay was 0.05 (OD_600_).

### Bacterial viability assays

Bacteria were grown in the nutrient-rich medium in conditions described above to an OD_600_ of 0.6–1.0. Bacteria were pelleted and washed twice in PBS-T. Washed cells were resuspended in 10 mL of MMAT minimal medium starting at OD_600_ of 0.05 for *M. smegmatis* and *M. abscessus* or 0.1 for Mtb. At the indicated time points, cultures were mixed by pipetting and serial diluted in PBS-T. For *M. smegmatis* and *M. abscessus*, 50 µL of samples were plated on LB agar plates and incubated at 37°C for 3–4 days. For *M*. *tuberculosis*, 50 µL of samples were plated on 7H10 OADC (with 100 µg/mL cycloheximide) agar plates and incubated at 37°C for 3–4 weeks.

### Bacterial lysis assay

Prior to the assay, *M. smegmatis* was transformed with pSMT1 plasmids expressing either Envy1-GFP or mCherry mycobacterium codon-optimized fluorescent protein genes (21). *M. smegmatis* strains were cultured under hygromycin antibiotic selection in MMAT minimal medium as described above buffered to pH 7.0 with either 100 mM MOPS or 100 mM sodium citrate and supplemented with 10 mM glycerol at a starting OD_600_ of ~0.6. At designated time points, 1 mL of samples were taken and the optical density was measured. The 1 mL of samples were then gently pelleted at 1,000 rpm and supernatants were removed and re-centrifuged to remove cells. Supernatants were then filtered using 0.20-µm filter, and culture filtrates were placed into clear bottom black wall 384-well plates. The relative fluorescence intensity (RFI) was measured using a Perkin Elmer plate reader for Envy1-GFP (Ex: 488 nm/Em: 515 nm) or mCherry (Ex: 580 nm/Em: 610 nm). Either MOPS or sodium citrate buffered cell-free medium was used for background subtraction. The assays were performed in biological duplicate and fluorescent readings were performed in technical triplicate for each sample. To ensure contaminating bacteria were not present in culture filtrates, following fluorescent readings, filtrates were plated onto LB agar plates and incubated for at least 5 days at 37°C. No bacteria were detected for any of the samples reported.

Cell lysis is reported as a ratio of two A.U. measures (RFI/OD_600_) and therefore unitless. Statistics comparing the ratio of RFI/OD between MOPS and sodium citrate buffered samples were calculated using a two-way ANOVA.

### Transcriptional profiling using RNAseq

*M. smegmatis* was cultured starting in 30 mL of MMAT medium supplemented with 10 mM glycerol buffered to pH 7.0 with 100 mM MOPS to pre-adapt cells. *M. smegmatis* was cultured to an OD_600_ of ~0.6 before being washed twice in MOPS-buffered MMAT medium and then split into 10 mL cultures of MMAT medium supplemented with 10 mM glycerol buffered to pH 7.0 with either 100 mM MOPS or sodium citrate in T25 flasks. Cultures were incubated for 3 h standing at 37°C before RNA was extracted and sequenced as previously described (19). RNAseq data were analyzed using the SPARTA software package (22).

### Cation supplementation

*M. smegmatis* cells were cultured in sodium citrate-buffered MMAT as described above. Sodium citrate cultures were supplemented with 100 µM of Fe_2_Cl_3_, MgCl_2_, MgSO_4_, CaCl_2_, ZnSO_4_, MnCl_2_, or CuSO_4_. At indicated time points, 50 µL of samples were serial diluted, plated on LB agar plates, and incubated as described above.

### Transposon mutagenesis

Transposon mutagenesis was performed as previously described (23). Briefly, φMycoMarT7 phage was harvested from *E. coli* and used to transfect *M. smegmatis* cells resulting in a ~30,000 mutant library selected for using 20 µg/mL kanamycin. Mutants were collected and the library was stored at −80°C. On the day of the experiment, the libraries were inoculated into 100 mL of MMAT medium buffered with 100 mM sodium citrate and supplemented with 10 mM glycerol in a T150 standing flask and cultured for 8 days at 37°C. Following incubation, cells were pelleted, washed in PBS-T, plated on 7H10 OADC agar plates with kanamycin, and incubated at 37°C. Colonies were picked and grown in a liquid medium with kanamycin. Transposon insertions were sequenced using inverse PCR (24). Sequences were mapped to the *M. smegmatis* genome to identify insertion sites. For complementation studies, the *mgtE* or MSMEG_2788 genes were cloned in the replicating pVV16 vector downstream of the constitutive *hsp*60 promoter.

### MagFura2-(AM) assay

*M. smegmatis* cells were labeled with the Mag-Fura2 (AM) fluorophore using a previously described protocol (25) with some modifications. Wild-type (WT) or the Tn:*mgtE M. smegmatis* strains were cultured to exponential growth (OD 0.6–1.0) in LB with 0.05% Tween-80. Approximately 6 × 10^9^ cells (OD_600_ of 0.6 = 1 × 10^8^ cells/mL) were harvested from overnight cultures and washed twice in 6 mL of 10 mM HEPES (pH 7.4) with 0.9% saline (HEPES-saline) and resuspended in 1 mL of HEPES-saline; 50 µg of MagFura2-AM was dissolved into 32.4 µL of dimethyl sulfoxide and 16 µL was added to the 1 mL of cell suspension along with 20 µM Pluronic F-127. Cells were incubated for 3 h shaking at 37°C shaking at 120 rpm. Cells were then pelleted and washed twice with 6 mL of PBS-T. Washed cells were resuspended in 6 mL of 0.9% saline with 0.05% tyloxapol and incubated at 37°C shaking at 120 rpm for an additional 30 min to allow for complete hydrolysis of AM ester. Cells were then pelleted and washed twice in PBS-T and resuspended to 3 × 10^8^ cells/mL in MMAT buffered with 100 mM MOPS or sodium citrate and supplemented with 10 mM glycerol. Cells were aliquoted into 384-well plates, and Mg^2+^ or Ca^2+^ was added to each well at the concentrations described in the text. Plates were read using a Perkin Elmer plate reader. Excitation and emission were as previously described (25) (high Mg^2+^, Ex: 380 nm/Em: 510 nm) or (low Mg^2+^, Ex: 340 nm/Em: 510 nm). The ratios were represented as the emission ratio (380 nm/340 nm). Experiments were conducted in biological duplicate and repeated at least twice.

### Ethylenediaminetetraacetic acid tolerance assay

*M. smegmatis* was grown to exponential growth in LB with 0.05% tyloxapol at 37°C in T25 standing flasks. Samples were washed twice in PBS-T and resuspended in pH 7.0 MMAT medium with 50 or 75 mM ethylenediaminetetraacetic acid (EDTA) and supplemented with 10 mM glycerol at a starting OD_600_ of 0.05. At exposure and 6 days postexposure, cultures were serial diluted in PBS with 0.05% tyloxapol; 50 µL of samples were plated on LB agar plates for *M. smegmatis* and incubated at 37°C for 3–4 days. The experiment was repeated twice.

### SDS sensitivity assay

*M. smegmatis* was grown in LB with 0.05% Tween-80 at 37°C, standing, to exponential phase. Cells were washed twice in PBS-T and resuspended at a starting OD_600_ of 0.05 in pH 7.0 MMAT medium with 100 mM sodium citrate and 10 mM glycerol. Samples were taken at time 0 and after 3 h post-incubation; they were serial diluted in PBS-T and 50 μL aliquots were plated on LB agar plates. Cultures were then washed twice in PBS-T and resuspended in pH 7.0 MMAT medium with 0.1% sodium dodecyl sulfate and 10 mM glycerol. Cultures were incubated at 37C for 4 h; 100 μL of samples were serial diluted and plated on LB agar. All agar plates were incubated for 3–4 days at 37°C and colony forming units (CFUs) were enumerated. The experiment was repeated twice.

### Meropenem-sensitivity assay

*M. smegmatis* was grown in 7H9 with OADC and 0.05% (vol/vol) tween80 to an OD_600_ of 0.6–1.0. Bacteria were pelleted by centrifugation (2,000 rpm for 10 min) and washed twice with PBS-T. Washed cells were resuspended in 30 mL MMAT minimal medium containing 0.05% (vol/vol) tyloxapol and buffered to pH 7.0 with either 100 mM MOPS or sodium citrate at a starting OD_600_ of 0.1. Resuspended cultures were aliquoted into 10 mL cultures in T-25 vented tissue culture flasks. Flasks were supplemented with meropenem for a final concentration per flask of 25 µM, 45 µM, or the vehicle (water) control. Samples were incubated for 4 or 24 h, shaking at ~120 rpm. All samples had biological triplicates. After incubation, cultures were serial diluted and plated for CFUs on LB agar. Plates were incubated at 37°C for 3–4 days before CFU enumeration. All plated sample dilutions had technical duplicates. The experiment was repeated at least twice.

### Osmoprotectant supplementation assay

*M. smegmatis* was cultured as described for the cell viability assay. However, after washing the cells, the cells were resuspended in MMAT with 10 mM glycerol buffered with MOPS or sodium citrate and supplemented with 200 or 20 mM of betaine, ectoine, or proline. The assay was conducted in biological duplicate and repeated twice with similar results. Comparison between groups was carried out using a two-way ANOVA by Fisher’s least significant difference (LSD) test.

## RESULTS

### Concentrated sodium citrate kills *M. smegmatis*

**Fig 1:**
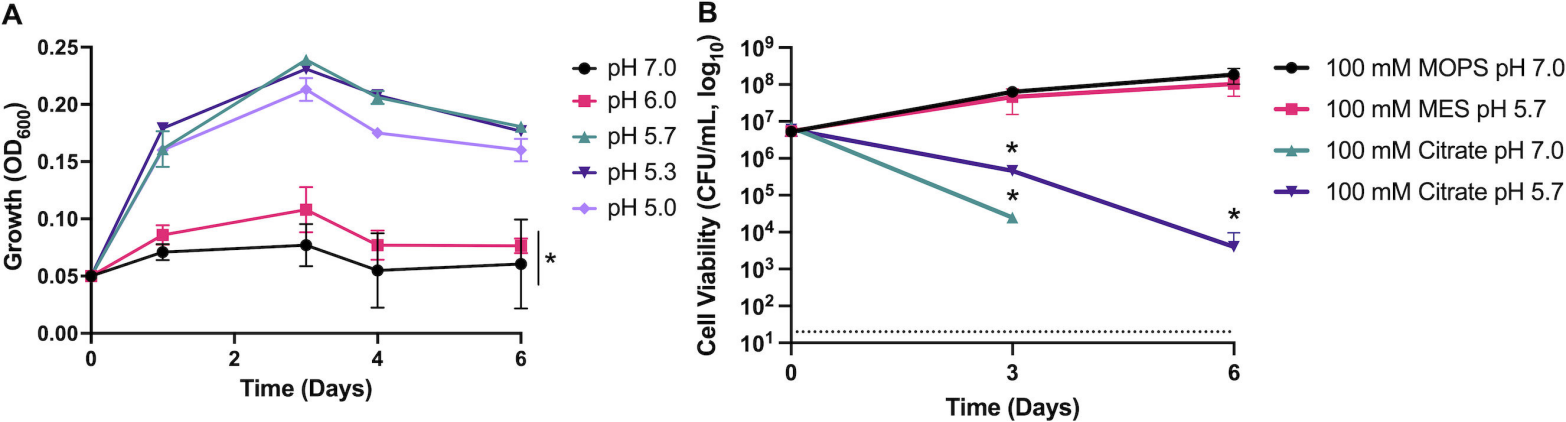
Sodium citrate kills *M. smegmatis*. (**A**) Growth of *M. smegmatis* cultured in MMAT minimal medium buffered with 100 mM sodium citrate to pH 7.0, 6.5, 5.7, 5.3, or 5.0. The minimal medium was supplemented with 10 mM glycerol. At day 6, cultures grown at pH 7.0 and 6.0 have significantly reduced growth compared to cultures grown at pH 5.7, 5.3, and 5.0 s (*P < 0.05, unpaired *t*-test). (**B**) *M. smegmatis* was cultured in MMAT minimal medium supplemented with 10 mM glycerol and buffered with 100 mM MOPS (pH 7.0), MES (pH 5.7), or sodium citrate (pH 5.7 or 7.0) and enumerated for CFU/mL on LB agar. For *M. smegmatis* cultured in 100 mM sodium citrate (pH 7.0), no colonies were isolated from day 6 samples. The figure represents results typical of these experiments. Experiments were conducted in triplicate and repeated at least twice. The dotted line indicates the limit of detection (20 CFU/mL). Bacterial viability is significantly lower in sodium citrate medium at days 3 and 6, relative to the MOPS control (*P < 0.05, unpaired *t*-test).

Previous studies into the metabolic adaptation of Mtb to acidic environments found that Mtb enters into a state of non-replicating persistence when cultured in a minimal medium (MMAT) buffered to pH 5.7 and provided specific sole carbon sources (18, 19). This phenotype is genetically regulated and is dependent on both pH and carbon source availability (18, 19). When cultured in MMAT at neutral pH (7.0), Mtb can grow on carbon sources such as glucose and glycerol (Fig. S1a) (18, 19). However, at acidic pH, Mtb will slow its metabolism and enter into a state of non-replicating persistence (Fig. S1b) when grown on these same carbon sources (19). This process can be reversed through supplementation with “permissive” carbon sources such as acetate or pyruvate (19). However, the slow doubling time of Mtb acts as a limiting factor for studying this phenotype. To overcome this limitation, we sought to recapitulate this phenotype in the rapidly growing mycobacterium species *M. smegmatis*. However, we found no significant difference in the growth *of M. smegmatis* cultured in a minimal medium buffered to neutral (100 mM MOPS, pH 7.0) or acidic pH (100 mM MES, pH 5.7) (Fig. S1c). This finding is consistent with prior findings (19, 26), where *M. smegmatis* is more tolerant to acidic pH than Mtb (26) and we hypothesized *M. smegmatis* may require a more acidic environment to induce growth arrest. To achieve this, we buffered a minimal medium with 100 mM sodium citrate (SC) in a pH range from pH 7.0 to pH 5.0. Counter to our hypothesis, in this medium, we observed that the growth of *M. smegmatis* was dramatically reduced when the pH approached neutral rather than acidic pH (Fig. 1A). Further investigation indicated that SC medium led to decreased viability of *M. smegmatis* at both pH 5.7 and 7.0, but was more pronounced at pH 7.0 (Fig. 1B). This phenotype was not observed in SC medium lacking a carbon source (i.e., glycerol), suggesting that active replication was required (Fig. S1c). This phenotype was not observed in Mtb, where viability was not affected by sodium citrate at either neutral or acidic pH (Fig. S1a and b). In contrast to *M. smegmatis*, Mtb growth was slightly reduced at neutral pH when supplemented with glycerol, and fully prevented when supplemented with glucose (Fig. S1a). We also tested this phenotype in the related rapidly growing mycobacterial species *M. abscessus*, which was insensitive to SC medium compared to MOPS medium (Fig. S1d). We found that this species-specific phenotype interesting and decided to investigate it, believing that it could shed light on the physiology of the model organism *M. smegmatis* and provide insights into distinct adaptations between pathogens and non-pathogens.

### Sodium citrate induces cell lysis in *M. smegmatis*

**Fig 2:**
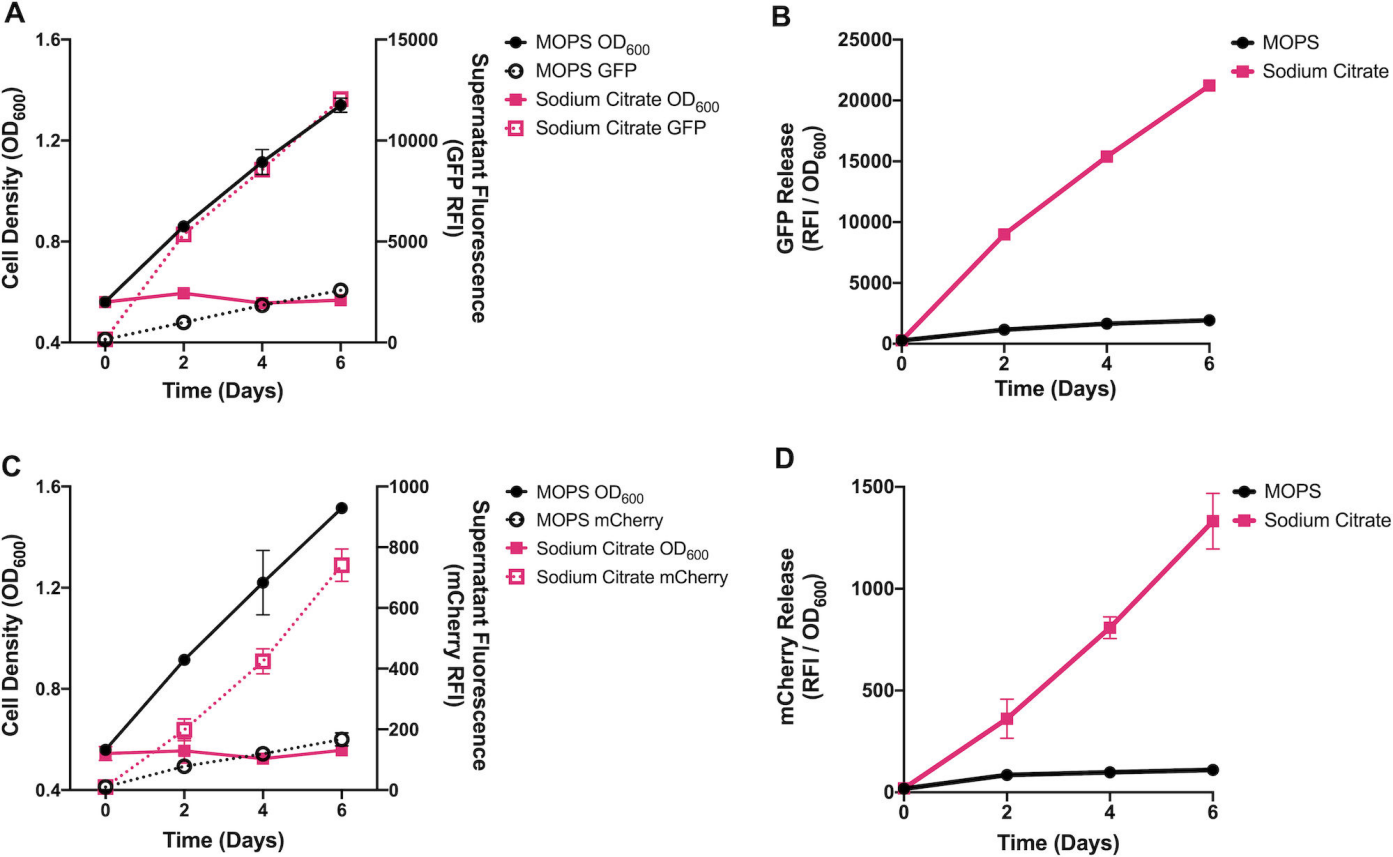
Sodium citrate induces *M. smegmatis* cell lysis. *M. smegmatis* strains expressing either GFP of mCherry were cultured in MOPS or sodium citrate buffered medium supplemented with 10 mM glycerol. (**A and C**) At indicated time points, 1 mL of sample was taken and measured for OD_600_ (solid lines). (**A and C**) Culture filtrates were then measured for fluorescence (GFP [Ex: 488 nm/Em: 515 nm] or mCherry [Ex: 587 nm/Em: 610 nm]) (dotted lines). (**B and D**) Relative RFI/OD_600_ measurements were made for each time point. The experiment was conducted in biological duplicate and fluorescent samples were measured in technical triplicate per sample. Data points indicate the mean of each sample. Statistics comparing the ratio of RFI/OD between MOPS and sodium citrate buffered samples were calculated using a two-way ANOVA. Starting on day 2, cell lysis measurements were statistically different between MOPS and sodium citrate cultures (*P* < 0.0001) in both assays.

Citrate is a carbon source for many bacteria and acts as an intermediate of the tricarboxylic acid cycle. However, several studies have indicated that *M. smegmatis* does not import citrate in conditions similar to the ones reported here (27, 28), suggesting that citrate was inducing stress on the cell envelope. A previous study by Nagaoka and colleagues demonstrated that sodium citrate induced cell swelling and bursting in *Streptococcus pneumoniae* (29). Consistent with these observations, we observed a decrease in the opacity of the bacteria grown in SC cultures during the cell viability assays described above indicating that cell lysis may have been occurring. To test for cell lysis, we used a combined OD_600_ and fluorescent protein release assay based on work by Sharma and colleagues (30). For this assay, we cultured *M. smegmatis* expressing either GFP or mCherry in SC or MOPS medium. We hypothesized that cultures undergoing cell lysis would release fluorescent protein into the supernatant (30). Consistent with this hypothesis, the fluorescence of culture filtrates from SC-cultured bacteria increased over time (Fig. 2A and C). While fluorescence increased, the OD_600_ of *M. smegmatis* did not change significantly during the experiment indicating there was no bacterial growth (Fig. 2). By comparison, the OD_600_ of MOPS cultured *M. smegmatis* increased dramatically over time but the filtrate fluorescence only increased marginally (Fig. 2) and was likely due to spontaneous cell lysis during cell replication. The differences in the amount of cell lysis were highlighted by the filtrate fluorescence to cell density ratio (RFI / OD_600_) in SC compared to the MOPS culture (Fig. 2B and D) which were significantly different (*P* < 0.0001) by day 2 post-inoculation. The results of this assay support that SC induces cell lysis.

### Sodium citrate induces chelation and hyperosmotic stress

**Fig 3:**
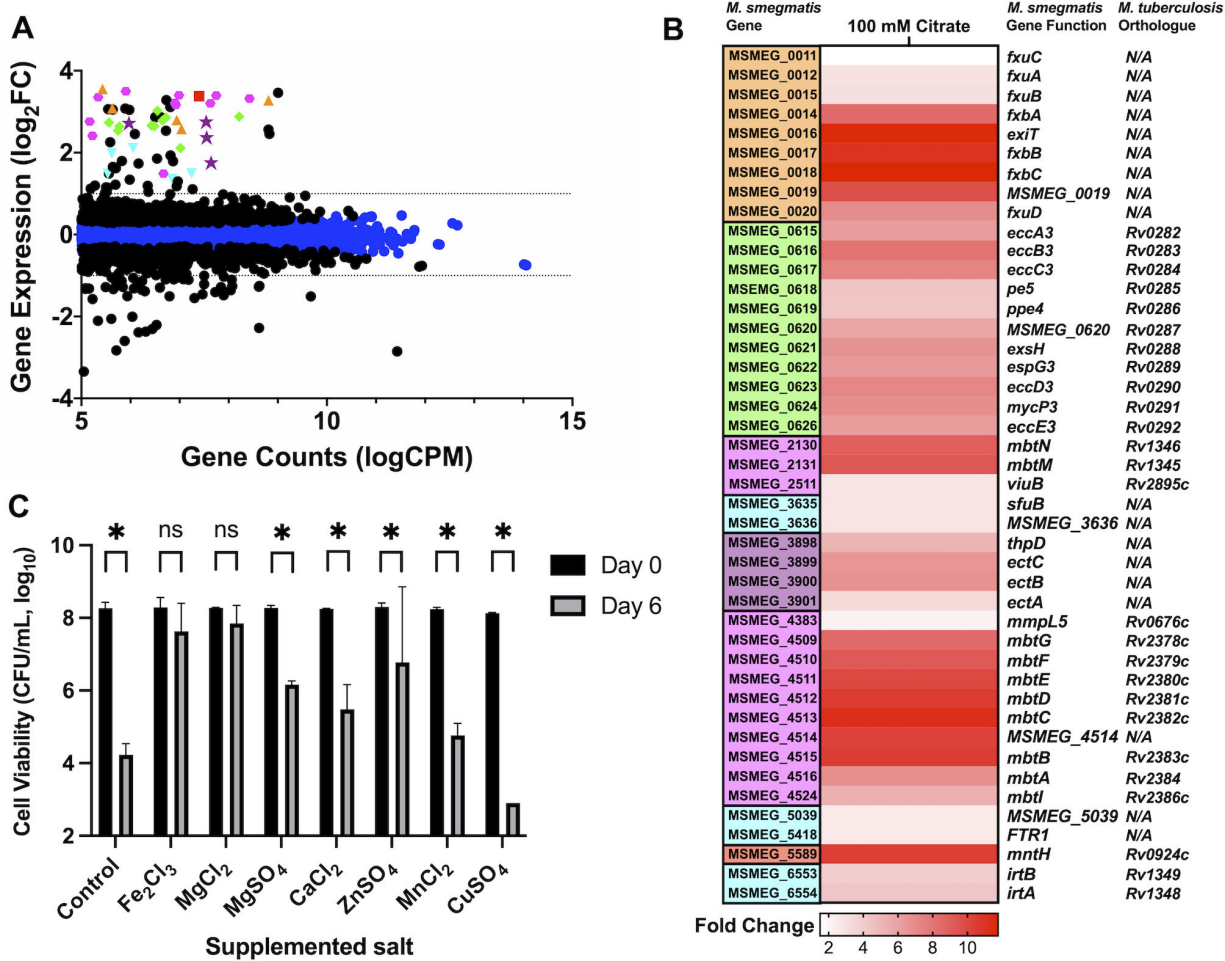
Sodium citrate induces chelation and osmotic stress. (**A**) RNAseq scatter plot of genes upregulated in *M. smegmatis* cultured citrate-buffered medium relative to MOPS-buffered controls. ● indicates significantly differentiated expressed genes (*q* < 0.05). The dotted lines indicate log_2_ fold changes of 1 or −1. Genes in specific metabolic pathways are highlighted and include exochelin biosynthesis (orange square), mycobactin biosynthesis (pink hexagon), ESX-3 secretion system (green diamonds), iron interacting genes (blue inverted triangles), ectoine biosynthesis (purple stars), and manganese transport (red square). (**B**) Heat map of genes differentially expressed in RNAseq scatter plot. Genes are color coordinated with scatter plots. Gene function annotations are included as well as M. *tuberculosis*-predicted homology. (**C**) Cell viability of *M. smegmatis* cultured in sodium citrate medium supplemented with 100 µM of Fe_2_Cl_3_, MgCl_2_, MgSO_4_, CaCl_2_, ZnSO_4_, MnCl_2_, or CuSO_4_. The experiment was conducted in duplicate and repeated at least twice. The error bars indicate the SD of the mean, *P < 0.05, ns, not significant unpaired *t*-test.

Based on the results of the cell viability and lysis assays, we understood that citrate induced cell death through cell lysis and was dependent on a carbon source; however, the specific mechanisms driving killing remained unclear. To gain a better understanding of what stress *M. smegmatis* sensed and was responding to, we used RNAseq-based transcriptional profiling of *M. smegmatis* cultured in either MOPS or SC-buffered minimal medium. To ensure that transcriptional profiles were not obscured by genes associated with cell death, we isolated RNA samples at an early time point (3 h). RNA was then sequenced by Illumina-based high-throughput sequencing and analyzed using the SPARTA software package (22). The resulting transcriptional profiles (Fig. 3A and B; Table S1) identified 72 and 54 genes upregulated or downregulated, respectively, in the sodium citrate profile compared to MOPS control (>2-fold, *q* < 0.05, Fig. 3A and B; Table S1). In the transcriptional profiles, we identified the signature profile of iron starvation including the siderophores exochelin (31 – 33) and mycobactin (33 – 37) and their biosynthesis and transport (33, 38, 39) (*MSMEG_0014*, *MSMEG_0018-MSMEG_0022*, *MSMEG_2130-MSMEG_2131*, *MSMEG_2511, MSMEG_4509-MSMEG_4516, MSMEG_4524,* and *MSMEG_4383*) (Fig. 3A and B). We also identified genes required for the type VII ESX-3 secretion system (*MSMEG_0615-MSMEG_0626*) as upregulated >2-fold (Fig. 3A and B). ESX-3 is required for siderophore import (40 – 42) and is involved in iron and zinc homeostasis (43, 44). In addition to the siderophore-based iron acquisition, mycobacteria can also import free iron through the IrtAB transporter (45 – 47). Genes for these transporters as well as other iron-interacting proteins (33, 34, 45, 46) were also upregulated in our RNAseq profile (*MSMEG_3635, MSMEG_3636, MSMEG_5039, MSMEG_5418, MSMEG_6553,* and *MSMEG_6554*) (Fig. 3A and B). We also identified upregulation of *MSMEG_5589* and *MSMEG_2607-MSMEG_2608* which are predicted to encode manganese and cobalt transporters, respectively (Fig. 3A and B) (15). Citrate can be used as a metal chelator and is often used in food production for preventing microbial growth (48). The profiles generated here support a model in which citrate was starving the bacteria of essential metal cations.

To test the hypothesis that citrate was acting as a metal chelator, we supplemented the SC-buffered medium with 100 µM of Fe_2_Cl_3_, MgCl_2_, MgSO_4_, CaCl_2_, ZnSO_4_, MnCl_2_, or CuSO_4_. Consistent with our hypothesis, no significant killing by sodium citrate was observed following supplementation with Fe_2_Cl_3_ and MgCl_2_ (Fig. 3C). While moderate rescue was observed in the presence of MgSO_4_, CaCl_2_, ZnSO_4_, and MnCl_2_ compared to the control, significant killing still occurred in these treatments. Supplementation with copper did not lead to cell viability rescue and instead enhanced killing likely due to the bactericidal effects of Cu through reactive oxygen species production (49). The results of this assay support a model where *M. smegmatis* is starved of metal ions during SC culture. To further confirm killing was driven by metal chelation, we examined viability in response to another chelator EDTA. When treated with 75 mM EDTA or 100 mM SC, we observed a similar ~2 log of killing of *M. smegmatis* (Fig. S2A). Together, these data support that the killing of *M. smegmatis* by SC is driven, in part, by the chelation activity of sodium citrate.

In addition to metal starvation genes, we also identified genes involved in hyperosmotic stress as upregulated >2-fold (Fig. 3A and B). Included in this list of genes was the osmotic stress regulatory gene *oprA* (*MSMEG_0586*) which is upregulated in Mtb in response to high salinity (17). We also identified genes involved in the biosynthesis of the compatible solute ectoine *ectA-C* and *thpD* (*MSMEG_3898 – MSMEG_3901*) (Fig. 3A and B) (13). In addition, we saw an increase in the expression of the sodium/proline importer *putP* (*MSMEG_5303*) which is involved in osmotic stress adaptation in *Staphylococcus aureus* (50). We hypothesize that these genes are induced in response to the high sodium included as the counterion to citrate used in our buffering system. This finding is consistent with a previous study that observed both *oprA* and ectoine biosynthesis genes are upregulated in high sodium (13, 17). Given the observed cell lysis in SC and differential regulation of osmotic stress genes, we hypothesized that this treatment may sensitize the bacteria to a cell envelope targeting agents, such as SDS treatment. However, when treated with 0.1% SDS for 4 h, no difference in viability was observed in the MOPS or SC-treated cells (Fig. S2B). We next examined whether the bacteria are more sensitive to an antibiotic targeting the integrity of the cell wall. *M. smegmatis* was treated with 25 or 45 µM meropenem. In the MOPS medium, we observed significant killing of *M. smegmatis* by meropenem; however, no additional killing was observed in bacteria cultured in the SC medium (Fig. S3A). Together, these data demonstrate that SC does not sensitize *M. smegmatis* to cell envelope-targeting agents.

The results of the transcriptional profiling suggested that SC medium induced both chelation and hyperosmotic stress in *M. smegmatis*. However, these results did not fully explain the bactericidal and cell lysis effects observed. Mycobacteria can survive when starved of exogenous cations in culturing conditions like PBS (Fig. S3B). In addition, deletion of ectoine biosynthesis genes in *M. smegmatis* leads to decreased growth but not cell death in high-saline solutions (13). Therefore, we sought to investigate this phenotype further to gain a better understanding of why SC medium induced cell death.

### A genetic selection identifies citrate-tolerant *M. smegmatis* mutants

**Fig 4:**
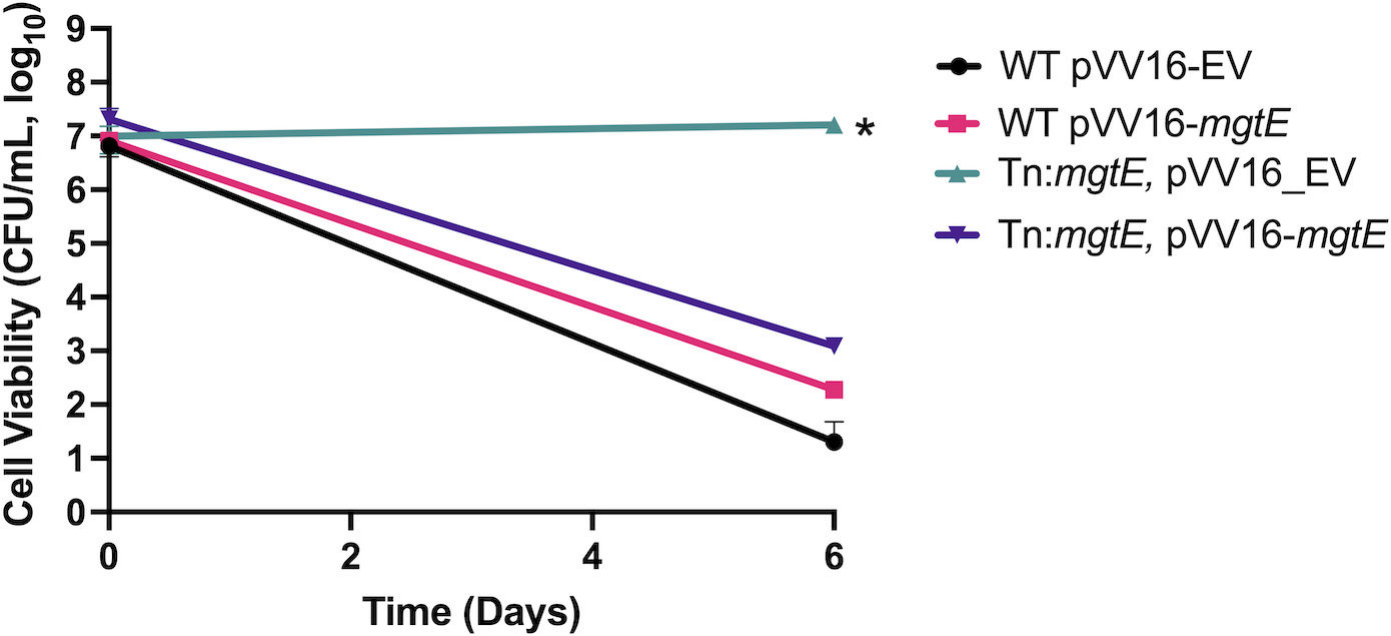
*M. smegmatis mgtE* transposon mutant is sodium citrate tolerant. Viability of WT, *mgtE* mutant, and complemented strains in MMAT minimal medium buffered with 100 mM sodium citrate (pH 7.0) and supplemented with 10 mM glycerol. The experiment was conducted in duplicate and repeated at least twice. The error bars indicate the SD from the mean. *P < 0.05 is significantly different from the WT and complemented strain in an unpaired *t*-test.

The results thus far suggested that SC induces a combination of chelation and osmotic stress, but the relative contribution of these stresses in killing was unknown. To gain further insights, we performed a genetic selection to identify mutants with resistance to killing by SC. Using the φMycoMarT7 phage transposon mutagenesis approach (51), we performed a genetic selection by culturing a ~30,000 transposon mutant library in SC medium for 8 days. Following incubation, surviving cells were plated on a solid nutrient-rich medium. After an outgrowth period of 4–5 days, individual colonies were picked and cultured in a fresh medium. Transposon insertion sites were then identified, including multiple independent insertions in genes of known and unknown function, and several singleton mutants (Table S2; Table 1). Mutants with multiple independent insertions included genes of seemingly unrelated pathways including *fadD6* (*MSMEG_5086, facl6*) involved in the very long-chain fatty acid synthesis and exogenous lipid acquisition (52, 53). Disruption of the magnesium importer *mgtE* (*MSMEG_6269*) was also found to confer SC tolerance. This seemed counterintuitive as Mg^2+^ supplementation led to cell viability rescue (Fig. 3C). We also identified independent insertions in two genes within a single operon encoding *MSMEG_6193* and *MSMEG_6195* predicted to encode homologs of *bagAB* (*Rv3679* and *Rv3680*) from Mtb (54, 55). Recently, *bagAB* was implicated in protecting Mtb from toxicity induced by glycerol metabolism and nitric oxide (55), a finding that was interesting as glycerol was included as our sole carbon source during the genetic selection. We also identified *MSMEG_2788,* a gene of unknown function, as well as *MSMEG_0973,* a predicted homolog to *Rv0528* of Mtb. Finally, we identified two insertions in *MSMEG_3184* encoding *treZ* involved in trehalose biosynthesis. In confirmation assays enumerating CFUs over time following incubation in SC medium, mutants in *mgtE,* MSMEG_2788, MSMEG_5086, and MSMEG_6195 exhibited >3 logs higher viability than the parental WT *M. smegmatis* (Fig. 4; Fig. S4). We successfully generated complementation strains for two of the mutants (*mgtE* and MSMEG_2788) and observed complementation for these genes (Fig. 4; Fig. S4). We observed similar tolerance of the *mgtE* mutant in response to killing by 75 mM EDTA (Fig. S2C). Overall, the selection identified several genes with multiple independent insertions, two of which we have complemented, suggesting that disruption of these genes drives the observed phenotype rather than an unidentified background mutation. Of note, none of the genes identified were differentially regulated in the RNAseq data (Table S1; Fig. 3A and B; Table S2; Table 1). Several of these genes are also conserved in *M. leprae*, an obligate intracellular species that has undergone extreme genomic reduction (15). Conservation of the genes identified in *M. leprae* suggests that these genes are involved in intracellular survival. The interpretation of these selection results was not initially clear as gene disruption was not biased to any particular metabolic pathway. To better understand the results of the genetic selection, we focused on genes that were best understood from the literature, namely *mgtE*, *fadD6,* and *treZ*.

**TABLE 1:** Sodium citrate-tolerant transposon mutants identified in a genetic selection

*MSMEG* gene	Gene name	Mtb homolog	*M. leprae* homolog	Insertion sites (nt)^*a*^	Gene function
*MSMEG_5086*	*fadD6*	*Rv1206*	*ML1062*	219, 1718	Very long-chain acyl-CoA synthetase
*MSMEG_6269*	*mgtE*	*Rv0362*	pseudo	824, 900	Magnesium transporter
*MSMEG_6193*	*bagA*	*Rv3679*	*ML2305*	747, 751	ATP-binding protein
*MSMEG_6195*	*bagB*	*Rv3680*	*ML2306*	314, 397, 715	ATP-binding protein
*MSMEG_0973*	*MSMEG_0973*	*Rv0528*	*ML2410c*	32, 721	Conserved membrane protein
*MSMEG_2788*	*MSMEG_2788*	*Rv2670c*	*ML1341*	310, 418, 980	ATP/GTP-binding integral membrane protein
*MSMEG_3184*	*treZ*	*Rv1562c*	pseudo	670, 675	Malto-oligosyltrehalose trehalohydrolase

^*a*^ nt—nucleotide, pseudo—predicted non-functional gene, Mtb—*M. tuberculosis.*

### Citrate does not chelate Mg^2+^ and Ca^2+^ from cytoplasm supporting a function at the cell envelope

MgtE is a magnesium-specific importer (56) and one of two Mg^2+^ transporters in mycobacteria along with CorA (57). Disruption of this gene providing tolerance seemed counter to a chelation stress model where Mg^2+^ supplementation leads to cell viability rescue (Fig. 3C). However, several studies have indicated that the cell envelope of *E. coli* and *Bacillus subtilis* serves as Mg^2+^ reservoirs and that Mg^2+^, along with Ca^2+^, stabilizes the bacterial cell wall (2 – 9). One model suggests that divalent cations are stored in the cell walls of bacteria bringing them closer to metal transporters in the cytoplasmic membrane (5, 8, 58). Little is known about the metal contents of the mycobacterium cell wall; however, mycobacteria do have a peptidoglycan layer (59) which, in *B. subtilis*, serves as a reservoir for metal cations (8, 9). The disruption of *mgtE* suggested that Mg^2+^ supplementation may lead to cell viability rescue by stabilizing the cell envelope rather than through Mg^2+^ import. This model is consistent with the inability of *M. smegmatis* to import citrate arguing against the chelation of cytoplasmic metals.

**Fig 5:**
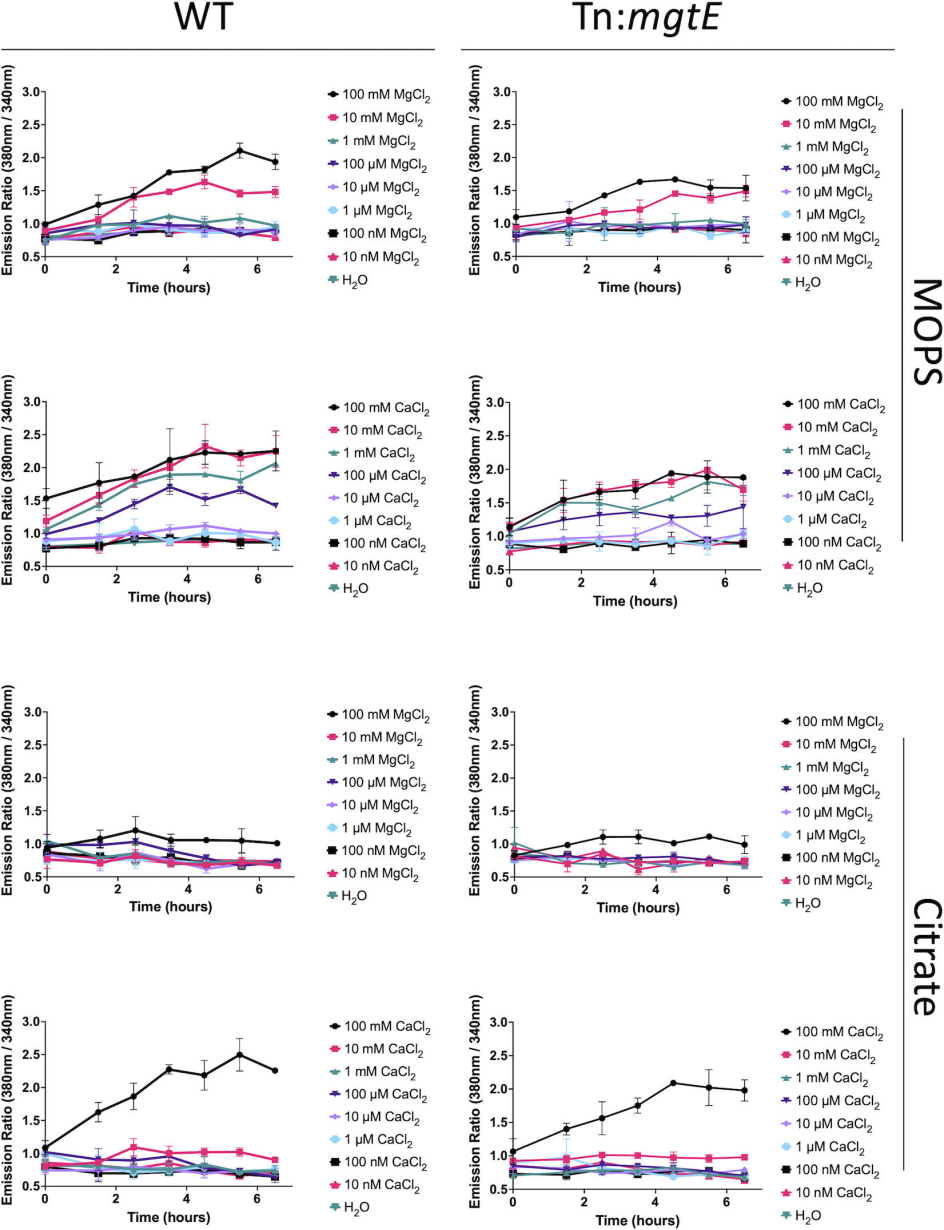
Sodium citrate prevents cation import in *M. smegmatis*. WT *M. smegmatis* or a Tn:*mgtE* was labeled with the cell-permeable Mag-Fura2 (AM) Mg^2+^/Ca^2+^ fluorophore and cultured in MOPS buffered or sodium citrate-buffered minimal medium. Cultures were supplemented with 100 mM to 10 nM concentrations of either Mg^2+^ or Ca^2+^. The ratio represents the RFI of cells measured at Mg^2+^ bound Mag-Fura2 (high Mg^2+^, Ex: 380 nm/ Em: 510 nm) relative to free Mag-Fura2 (low Mg^2+^, Ex: 340 nm/Em: 510 nm). The experiments were performed in biological duplicate and repeated at least twice. Error bars indicate the SD of the mean.

Currently, it is challenging to measure cell envelope metal ion concentrations in live cells. As an alternative method to test the hypothesis that citrate is acting at the cell envelope, we labeled the *M. smegmatis* cytoplasm with the cell-permeable magnesium fluorophore Mag-Fura2 (AM) (25). We hypothesized that if citrate was acting on the cell envelope, then supplemented cations will not be imported into the cell. We cultured Mag-Fura2 labeled cells in MOPS or SC-buffered medium with or without Mg^2+^ supplementation (100 mM to 10 nM). In addition to Mg^2+^, MagFura-2 can also detect Ca^2+^ ions (60). Since Ca^2+^ supplementation also rescued *M. smegmatis* cell viability (Fig. 3C), we also supplemented Mag-Fura2-loaded cells with Ca^2+^ (100 mM to 10 nM). Following Mg^2+^ and Ca^2+^ supplementation in MOPS-cultured cells, we observed a dramatic increase in the emission ratio (Ex: 380 nm/Ex: 340 nm) in a dose-dependent manner (Fig. 5A and C), demonstrating the potential for uptake of cations and defining a dynamic range for the assay. However, no increase in signal was observed for 100 µM or lower Mg^2+^ which rescued *M. smegmatis* in SC medium. By contrast, cells cultured in SC medium did not actively import either Mg^2+^ or Ca^2+^ except at the highest concentrations (100 mM) (Fig. 5). This included 100 µM Ca^2+^ which was able to rescue cell viability (Fig. 3C). Furthermore, SC medium did not result in a decrease in the emission ratio (Fig. 5) suggesting that citrate was not chelating Mg^2+^ or Ca^2+^ from the cytosol consistent with the inability of exogenous citrate to enter the cell (27, 28).

To determine whether the *mgtE* mutant had increased cation import due to an unidentified compensatory system, we also labeled the cytoplasm of the *mgtE* mutant with the Mag-Fura2 fluorophore and tested for cation import via dose response as we did for WT (Fig. 5). The results of the assay mirrored those observed in the WT suggesting that sodium citrate tolerance in the *mgtE* mutant is not due to changes in ion import. We noted a higher emission ratio following a high concentration of 100 mM Mg^2+^ supplementation with MOPS in WT (peak 2.1 at 5.5 h) compared to the *mgtE* (1.5 at 5.5 h) (Fig. 5, highlighted in Fig. S3) suggesting that the *mgtE* mutant is modestly impaired for Mg^2+^ import compared to WT. These data are consistent with the hypothesis that citrate is not acting on the cytoplasm and rather may function to chelate cell envelope cations which is predicted to weaken the cell envelope. These results are also consistent with the hypothesis that the *mgtE* mutant is tolerant of cell envelope chelation due to decreased cation import from the cell envelope, which may harden the cell envelope to osmotic stress.

### Specific compatible solute supplementation rescues *M. smegmatis* from killing by citrate

In addition to *mgtE*, disruption of *treZ* and *fadD6* also confers tolerance to killing by SC (Table 1). *treZ* is part of a two-gene pathway along with *treY* that metabolizes maltodextrin to form the disaccharide trehalose in a unidirectional enzymatic reaction (61). TreYZ forms one of three trehalose metabolic pathways in *M. smegmatis* along with TreS (62, 63) and OtsAB (63, 64). A previous study demonstrated that *treYZ* was the most upregulated in *M. smegmatis* as it entered dormancy resulting in cytosolic trehalose accumulation (65). However, the results of the genetic selection suggested that disruption of trehalose accumulation protected *M. smegmatis* from the SC medium. *fadD6* is one of several fatty acyl-CoA ligase (FACL) genes found in mycobacteria (15). In Mtb, FadD6 is involved in incorporating fatty acids into fatty acid esters such as triacylglycerol (TAG) (52, 53) which are stored as cytosolic lipid droplets during dormancy (66). As both *treZ* and *fadD6* are involved in intracellular substrate accumulation following environmental stress, we hypothesized that there was a cytosolic component to the sodium citrate phenotype.

**Fig 6:**
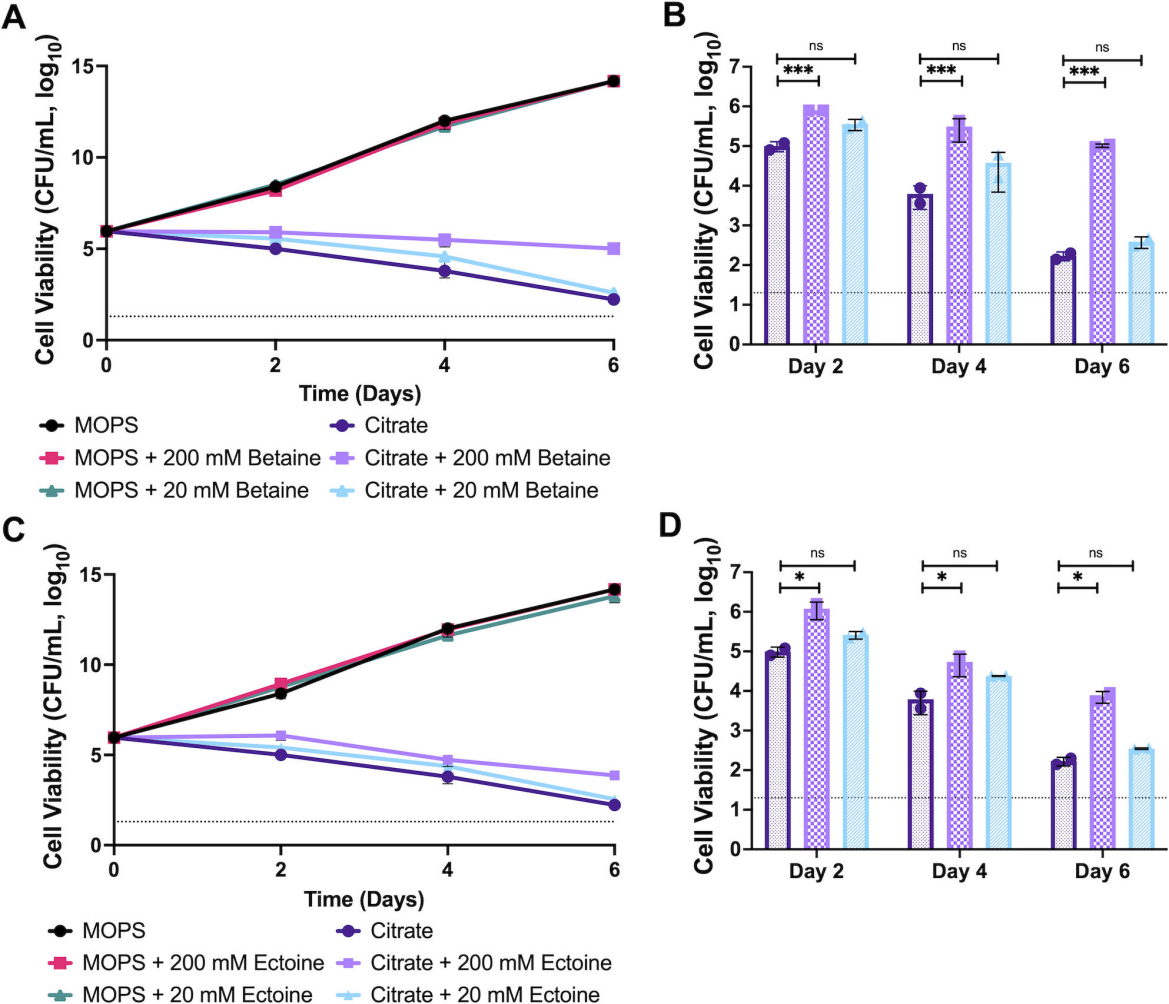
Osmolyte supplementation decreases *M. smegmatis* killing in sodium citrate. (**A and B**) *M. smegmatis* was cultured in MOPS or sodium citrate-buffered minimal medium supplemented with 20 mM or 200 mM betaine (**A**) or ectoine (**C**). (**B and D**) Bar graphs of cell viability of sodium citrate cultures (only) with or without osmoprotectant supplementation. Samples were compared using a two-way ANOVA with Fisher’s LSD test. The experiment was conducted in duplicate and repeated at least twice. Error bars indicate the SD of the mean and dotted lines indicate the limit of detection (20 CFUs/mL).

The RNAseq data suggested that *M. smegmatis* was adapting to hyperosmotic stress likely due to the high osmolyte (sodium and citrate) concentration in the SC medium (Fig. 3A and B). To test the hypothesis that *M. smegmatis* was experiencing hyperosmotic stress as part of killing by citrate, we supplemented both MOPS and SC cultures with 200 mM and 20 mM concentrations of the compatible solutes betaine, ectoine, or proline (Fig. 6A and C; Fig. S5a). Supplementation with both betaine and, to a lesser extent, ectoine at 200 mM, but not 20 mM, resulted in decreased killing of *M. smegmatis* in the SC medium (Fig. 6B and D). This reduction in killing was not due to decreases in growth as betaine and ectoine did not impact the growth of *M. smegmatis* in the MOPS medium (Fig. 6A and C). However, this reduction in killing was not observed in cultures supplemented with 200 mM or 20 mM proline (Fig. S5) suggesting that not all osmoprotectants can rescue *M. smegmatis* from sodium citrate-based killing. The relative concentration of osmotically active compounds is uncertain, given potential differences in their import, therefore, direct comparisons of the concentrations and activities are uncertain. Nevertheless, these data, along with the observed cell lysis, support a model in which cell death in the SC medium is enhanced through osmotic stress.

## DISCUSSION

In an attempt to recapitulate acidic pH-induced persistence in Mtb cultured in a minimal medium, we discovered a lethal SC phenotype in the model organism *M. smegmatis*. Transcriptional profiling using RNAseq indicated that SC induced cation chelation in *M. smegmatis*. Supporting this hypothesis, cation supplementation led to cell viability rescue. We also identified genes involved in osmotic stress adaptation, likely in response to the sodium ions in the solution. However*, M. smegmatis* can survive in both cation-depleted medium as well as high osmotic environments. To better understand this phenotype, we performed a forward genetic transposon mutagenesis screen in *M. smegmatis*. We identified SC-tolerant mutants with multiple independent transposon insertions in genes of seemingly unrelated metabolic pathways including *mgtE*, *fadD6,* and *treZ*. Using these mutants, we propose a model in which SC chelates cell envelope cations to weaken the cell wall while inducing hyperosmotic stress. The combination of these stresses results in the killing of *M. smegmatis* by cell lysis while tolerance to cell lysis can be achieved by either a strengthened cell wall or limiting hyperosmotic stress. In this model, WT *mgtE* imports Mg^2+^, and in the presence of SC chelating activity, there is less available Mg^2+^, so it is stripped from the cell envelope. *mgtE* mutation leads to SC tolerance through increased Mg^2+^ accumulation in the cell envelope resulting in increased cell envelope stability in the presence of citrate chelation. This model also proposes that *M. smegmatis* undergoes a stage of cell swelling due to responses needed to adapt to hyperosmotic stress including the synthesis of compatible solutes (e.g., ectoine) or possibly trehalose and lipid droplet accumulation. Over-accumulation of these compatible solutes could further weaken the cell envelope. Because there is no complementation data for the *treZ* and *fadD6* mutant strains, we lack direct evidence tying them to cell swelling or their function in modulating trehalose accumulation or lipid droplet accumulation in the examined conditions. Therefore, their role in this model remains speculative. However, based on our observed data and the identity of these genes, we speculate that this hyperosmotic stress is mitigated in the *treZ* and *fadD6* mutants, as they are hypothesized to not accumulate these solutes in the cytoplasm at levels comparable to the wild type. This model promises to serve as a starting point for additional investigation of the physiological roles of other genes identified in the transposon mutagenesis screen, several of which have no known function.

Cell death in SC is dependent on bacterial replication as media lacking a carbon source such as glycerol did not have the same bactericidal effects. This suggests that growth is required for the killing phenotype, possibly due to peptidoglycan remodeling and cell wall weakening associated with cell division. Consistent with this model, our transposon screen, which used glycerol as its sole carbon source, identified insertions in *MSMEG_6193* and *MSMEG_6195* which are homologous to Mtb *bagAB* (*Rv3679* and *Rv3680*) (54, 55). Recently, *bagAB* was implicated in glycerol and nitric oxide detoxification (55). Glycerol metabolism results in methylglyoxyl accumulation which can be toxic to mycobacteria (55, 67). These toxic effects result in decreased growth rate which would normally render *M. smegmatis* strains deficient in detoxification at a selective disadvantage compared to WT strains. However, our studies suggest that a higher growth rate is selected against in SC medium and may explain why genes involved in detoxification were identified in our screen. Further supporting this model, we identified insertion mutants in genes involved in central carbon metabolism (*MSMEG_2613* malate:quinone-oxidoreductase [*mqo*]) and electron transport chain biogenesis (*MSMEG_0973* predicted ResB cytochrome c biogenesis-like protein). Using this method, parallel carbon source screens can be developed to screen for genes required for specific metabolic pathways.

Citrate is a useful tool for studying chelation stress *in M. smegmatis* as exogenous citrate is not taken into the cell (27, 28). The chelation effects of citrate are dependent on the protonation state (pKa) of citrate (48). At lower pH levels, citrate becomes more protonated and the binding affinity for metals decreases (48). This would explain why we observed differences in the killing effect of *M. smegmatis* cultured in SC medium at pH 7.0 compared to pH 5.7 when citrate is in the −3 vs −2 charge states, respectively. This effect was not due to growth differences, as *M. smegmatis* grew to similar levels at both pH 7.0 and pH 5.7 in MOPS and MES-buffered medium. Cation supplementation rescued *M. smegmatis* cell viability at concentrations as low as 100 µM in SC medium. However, this was not due to cytoplasmic uptake as SC prevented Mg^2+^ and Ca^2+^ import at these concentrations, suggesting cation rescue may be due to cell envelope stabilization or saturating the chelation of citrate molecules. Consistent with these observations, Ca^2+^ supplementation has been reported to rescue a lethal LPS accumulation phenotype in *E. coli* through outer membrane stabilization (68). Future studies could seek to further characterize where these cations are accumulating and what role they play in mycobacterial cell envelope stability. Of note, the lethal phenotype we observed *in M. smegmatis* was not observed in Mtb or *M. abscessus*. These differences in outcome are likely growth rate independent, as the rapidly growing *M. abscessus*, which has a growth rate comparable to *M. smegmatis*, was completely insensitive to the inhibitory effects of SC medium. This suggests that the differences between species were due to other phenotypic differences, possibly the structures of the cell envelopes. A previous study highlighted the structural differences in the peptidoglycan between *M. smegmatis*, Mtb, and *M. lepra*e (59). Peptidoglycan is one of the major reservoirs for cell wall cations in *B. subtilis* (8, 9). Whether the differences in peptidoglycan between mycobacterial species play a role in the SC phenotypic outcome is unclear, nor is it clear whether the differences in peptidoglycan play a role in cation sequestration and further studies will be needed to address this hypothesis.

Finally, we observed that supplementation of the minimal medium with the osmoprotectants betaine, ectoine, but not proline, led to increased cell viability. The differences in outcome were striking as these three osmoprotectants have different metabolic outcomes in *M. smegmatis*. For one, betaine, which conferred the highest protection, is imported into Mtb to help protect from osmotic stress (14). This import occurs through the ProXVWZ import system, of which no homologs are predicted *for M. smegmatis* (14). Next, ectoine was able to partially rescue *M. smegmatis* from SC-based killing. *M. smegmatis* can synthesize ectoine and our RNAseq data demonstrated that the genes required for this biosynthesis were upregulated in SC-cultured cells. However, ectoine transport genes were not upregulated in these profiles suggesting *M. smegmatis* is not importing exogenous ectoine. Finally, the RNAseq profiles indicated that *M. smegmatis* cells may attempt to import proline based on the induction of the *putP* (*MSMEG_5303*) proline import gene. However, supplementation of SC medium with proline did not lead to cell viability rescue. These findings support adaptations to osmotic stress are needed in SC medium; however, the specific mechanisms remain to be resolved.

## Data Availability

The RNAseq data have been deposited in the GEO database (accession no. GSE180696).

## SUPPLEMENTAL MATERIAL

Supplemental Materials msphere.00358-23-s0001.pdf Supplemental figures and Table S2.

Table S1 msphere.00358-23-s0002.xlsx RNAseq data tables showing gene expression of *M. smegmatis* treated with sodium citrate, including lists of genes that are significantly differentially expressed.

ASM does not own the copyrights to Supplemental Material that may be linked to, or accessed through, an article. The authors have granted ASM a non-exclusive, world-wide license to publish the Supplemental Material files. Please contact the corresponding author directly for reuse.
