# Supplementary material for: A genetic selection for Mycobacterium smegmatis mutants tolerant to killing by sodium citrate defines a combined role for cation homeostasis and osmotic stress in cell death
Source: mSphere. 2023 Sep 8;8(5):e00358-23. doi: 10.1128/msphere.00358-23 (PMC10597346; doi:10.1128/msphere.00358-23)
Supplement: Supplemental Materials — Supplemental figures and Table S2. [file msphere.00358-23-s0001.pdf]

Supplemental Table 2. Identities of mutants tolerant to killing by citrate

Sample Name	MSMEG_Gene	Gene Insertion (nt)	Gene	Annotated Gene Function	TB Homolog	TB Gene
TN-85-40	MSMEG_0790	~430	MSMEG_0790	hydrolase, NUDIX family protein	Rv0413	mutT3
Tn-4-15	MSMEG_0973	32	HP	conserved membrane protein	Rv0528	Rv0528
TN-85-84	MSMEG_0973	721	HP	conserved membrane protein	Rv0528	Rv0528
TN-85-66	MSMEG_1004	160	MSMEG_1004	gene fragment	N/A	N/A
Tn-A1	MSMEG_1351	902	MSMEG_1351	cyclopropane-fatty-acyl-phospholipid synthase 1	Rv3392c	cmaA1
Tn-4-7	MSMEG_2579	277	MSMEG_2579	zinc metalloprotease	Rv2869c	rip
Tn-6-8	MSMEG_2613	394	mqo	malate:quinone-oxidoreductase	Rv2852c	Rv2852c
TN-85-3	MSMEG_2788	978	MSMEG_2788	ATP/GTP-binding integral membrane protein	Rv2670c	Rv2670c
TN-85-5	MSMEG_2788	310	MSMEG_2788	ATP/GTP-binding integral membrane protein	Rv2670c	Rv2670c
TN-85-69	MSMEG_2788	418	MSMEG_2788	ATP/GTP-binding integral membrane protein	Rv2670c	Rv2670c
TN-85-79	MSMEG_2788	980	MSMEG_2788	ATP/GTP-binding integral membrane protein	Rv2670c	Rv2670c
TN-85-57	MSMEG_2959	~301	gabT	4-aminobutyrate transaminase	Rv2589	gabT
TN-85-39	MSMEG_3184	~670	treZ	malto-oligosyltrehalose trehalohydrolase	Rv1562c	treZ
TN-85-54	MSMEG_3184	~675	treZ	malto-oligosyltrehalose trehalohydrolase	Rv1562c	treZ
Tn-C1	MSMEG_3236	242	MSMEG_3236	ABC-type amino acid transport system, permease component	N/A	N/A
TN-85-34	MSMEG_3638	758	MSMEG_3638	CBS domain protein	Rv1841c	Rv1841c
Tn-C3	MSMEG_4525	899	MSMEG_4525	putative oxygen-independent coproporphyrinogen III oxidase	Rv2388c	hemN
TN-85-2	MSMEG_4702	1152	MSMEG_4702	ABC-type transporter, permease components	Rv0102	Rv0102

TN-85-71	MSMEG_5086	1718	fadD6	very-long-chain acyl-CoA synthetase	Rv1206	fadD6
TN-85-79	MSMEG_5086	219	fadD6	very-long-chain acyl-CoA synthetase	Rv1206	fadD6
TN-85-26	Upstream of MSMEG_5201	upstream	Upstream of MSMEG_5201	regulatory protein GntR, HTH	N/A	N/A
TN-85-50	Upstream of MSMEG_5201	upstream	Upstream of MSMEG_5201	regulatory protein GntR, HTH	N/A	N/A
TN-85-31	MSMEG_5967	1222	MSMEG_5967	glucose methanol-choline oxidoreductase	Rv0492c	Rv0492c
TN-85-48	MSMEG_5967	1224	MSMEG_5967	glucose-methanol-choline oxidoreductase	Rv0492c	Rv0492c
TN-85-12	MSMEG_6193	747	MSMEG_6193	anion-transporting ATPase	Rv3679	bagA
TN-85-56	MSMEG_6193	751	MSMEG_6193	anion-transporting ATPase	Rv3679	bagA
Tn-2-2	MSMEG_6195	314	MSMEG_6195	ion-transporting ATPase	Rv3680	bagB
Tn-2-5	MSMEG_6195	397	MSMEG_6195	ion-transporting ATPase	Rv3680	bagB
Tn-2-6	MSMEG_6195	715	MSMEG_6195	ion-transporting ATPase	Rv3680	bagB
TN-85-38	MSMEG_6269	900	mgtE	magnesium transporter	Rv0362	mgtE
TN-85-74	MSMEG_6269	824	mgtE	magnesium transporter	Rv0362	mgtE
Tn-2-3	MSMEG_6404	213	glf	UDP-galactopyranose mutase	Rv3809c	glf

Supplemental Figures

Figure S1. Sodium citrate differentially effects the cell viability and growth of mycobacteria. A and **B**) *M. tuberculosis* cultured in MMAT minimal medium buffered with 100 mM MOPS (pH 7.0) (**A**), MES (pH 5.7) (**B**), or sodium citrate (pH 7.0 or pH 5.7) (**A** and **B** respectively) supplemented with 10 mM glucose or glycerol. **C**) Cell viability of *M. smegmatis* cultured in minimal medium buffered with 100 mM MOPS (pH 7.0), MES (pH 5.7), or sodium citrate (pH 7.0 or 5.7). Sodium citrate buffered cultures were supplemented with or without 10 mM glycerol. **D**) Cell viability of *M. abscessus* cultured in MMAT minimal medium buffered to pH 7.0 with 100 mM MOPS or sodium citrate and supplemented with 10 mM glycerol. Error bars indicate the s.d, of the mean.

Figure S2. Sensitivity of *M. smegmatis* to EDTA and SDS treatment. A. *M. smegmatis* cultured in MMAT minimal medium buffered with 100 mM sodium citrate or 75 mM (pH 7.0) show a similar significant decrease in survival. **B.** Treatment of *M. smegmatis* cultured in 100 mM MOPS or sodium citrate (pH 7.0) with 0.1% SDS. No differential sensitivity to killing by SDS was observed. **C. Tolerance of the *mgtE* mutant to killing by citrate and EDTA.** *M. smegmatis* WT, *mgtE* mutant and complemented strain were cultured in MMAT minimal medium buffered with 100 mM sodium citrate or 75 mM EDTA. The *mgtE* mutant had resistance to killing by both sodium citrate and EDTA. The experiments were conducted in duplicate and repeated at twice. The error bars indicate the s.d. of the mean, *, $p<0.05$, ns, not significant unpaired t-test.

Figure S3. A. *M. smegmatis* sensitivity to meropenem treatment. *M. smegmatis* was cultured in MMAT minimal medium buffered with 100 mM MOPS or sodium citrate (pH 7.0) and supplemented with 10 mM glycerol and treated with meropenem for 24 hours. Meropenem resulted in a significant decrease in survival in relative to the vehicle control in MOPS, but not in sodium citrate. The experiment was conducted in duplicate and repeated at least twice. The error bars indicate the s.d. of the mean, *, $p<0.05$, ns, not significant unpaired t-test. **B. *M. smegmatis***

is viable in the absence of environmental cations. *M. smegmatis* was cultured in phosphate buffered saline (pH 7.4) with 0.05% tween-80 and supplemented with 10 mM glycerol. **C. An *mgtE* mutant has decreased Mg^{2+} import relative to WT Msm.** WT *M. smegmatis* or a Tn:*mgtE* mutant were labeled with the cell permeable Mag-Fura2 (AM) Mg^{2+}/Ca^{2+} fluorophore and cultured in MOPS or sodium citrate buffered minimal medium pH 7.0. Cultures were supplemented with 100 mM Mg^{2+}. The ratio represents the RFI of cells measured at Mg^{2+} bound Mag-Fura2 (High Mg^{2+}, Ex: 380 nm/ Em: 510 nm) relative to free Mag-Fura2 (Low Mg^{2+}, Ex: 340 nm/ Em: 510 nm). The experiments were performed in biological duplicate and repeated at least twice. Error bars indicate the s.d. of the mean.

Figure S4. *M. smegmatis* transposon mutants are sodium citrate tolerant. WT and *MSMEG_2788* (**A**), *MSMEG_6195* (**B**) and *MSMEG_5086* (**C**), transposon mutant cell viability in MMAT minimal medium buffered with 100 mM sodium citrate (pH 7.0) and supplemented with 10 mM glycerol. a and b) Cell viability of complemented strains in MMAT minimal medium buffered with 100 mM sodium citrate (pH 7.0) and supplemented with 10 mM glycerol. The experiments were repeated twice with similar results.

Figure S5. Proline supplementation does not affect *M. smegmatis* viability in sodium citrate. A. *M. smegmatis* was cultured in MOPS or sodium citrate buffered minimal medium supplemented with 20 mM or 200 mM proline and enumerated for cell viability (CFU/mL) over six days. **B.** Cell viability of sodium citrate cultures with or without osmoprotectant supplementation. Samples were compared using a Two-way ANOVA with Fisher's LSD test. Error bars indicate the s.d. of the mean and dotted lines indicate the limit of detection (20 CFU/mL).

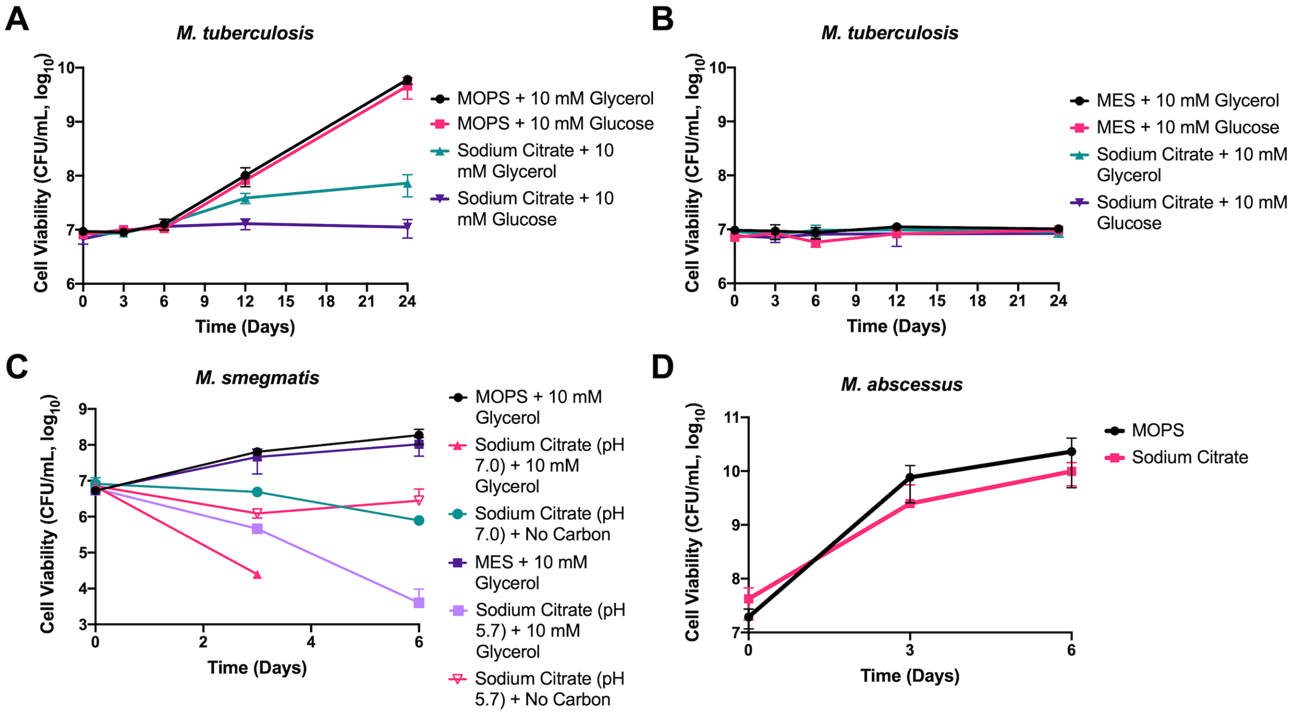

Supplemental Figure 1

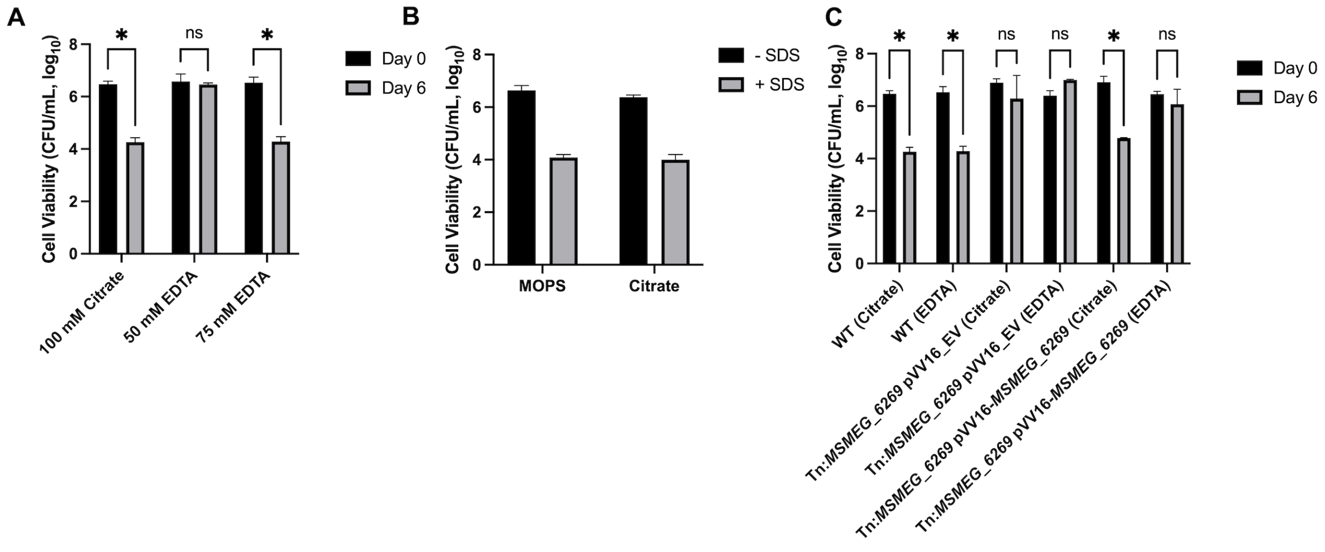

Supplemental Figure 2

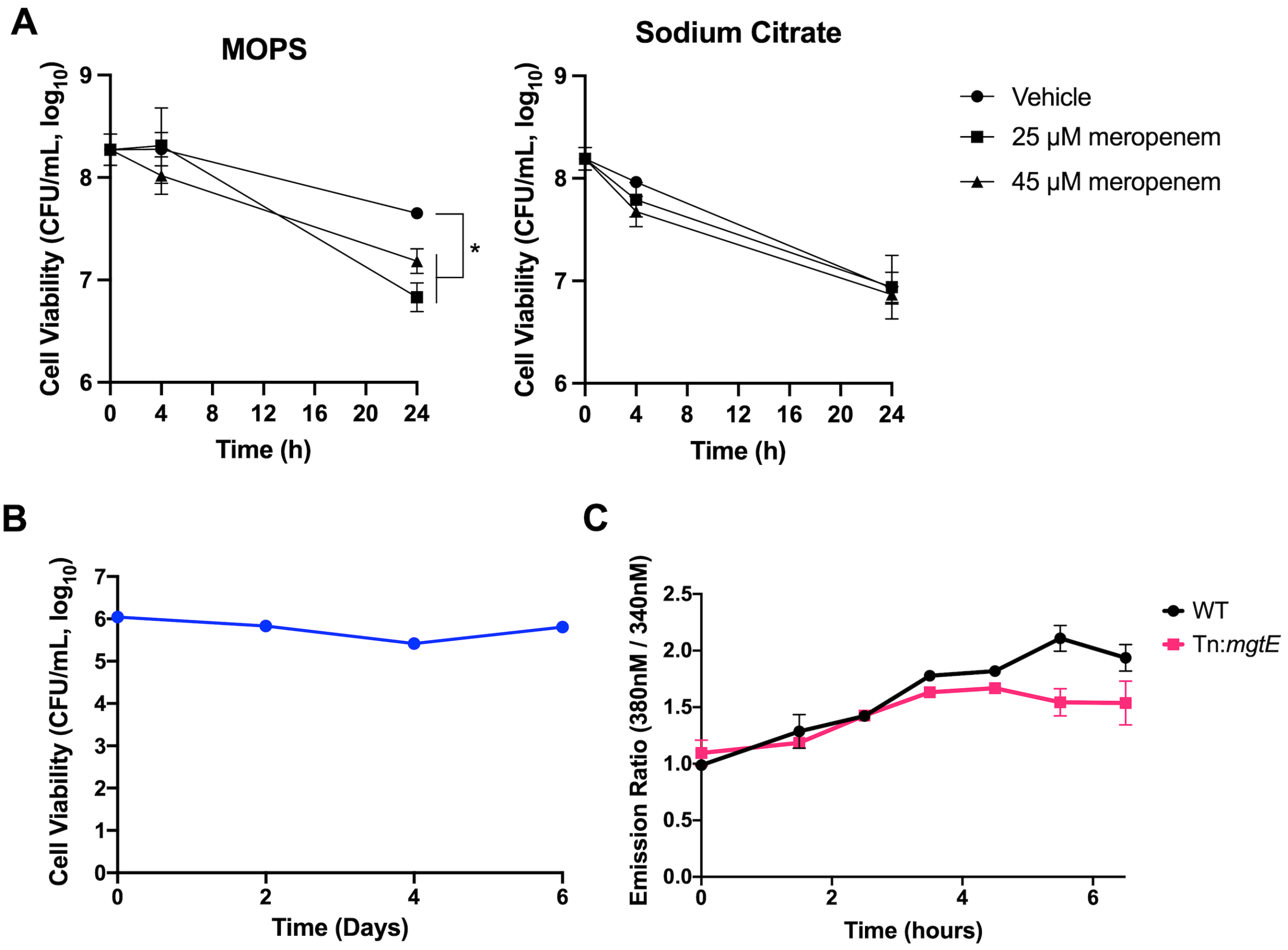

Supplemental Figure 3

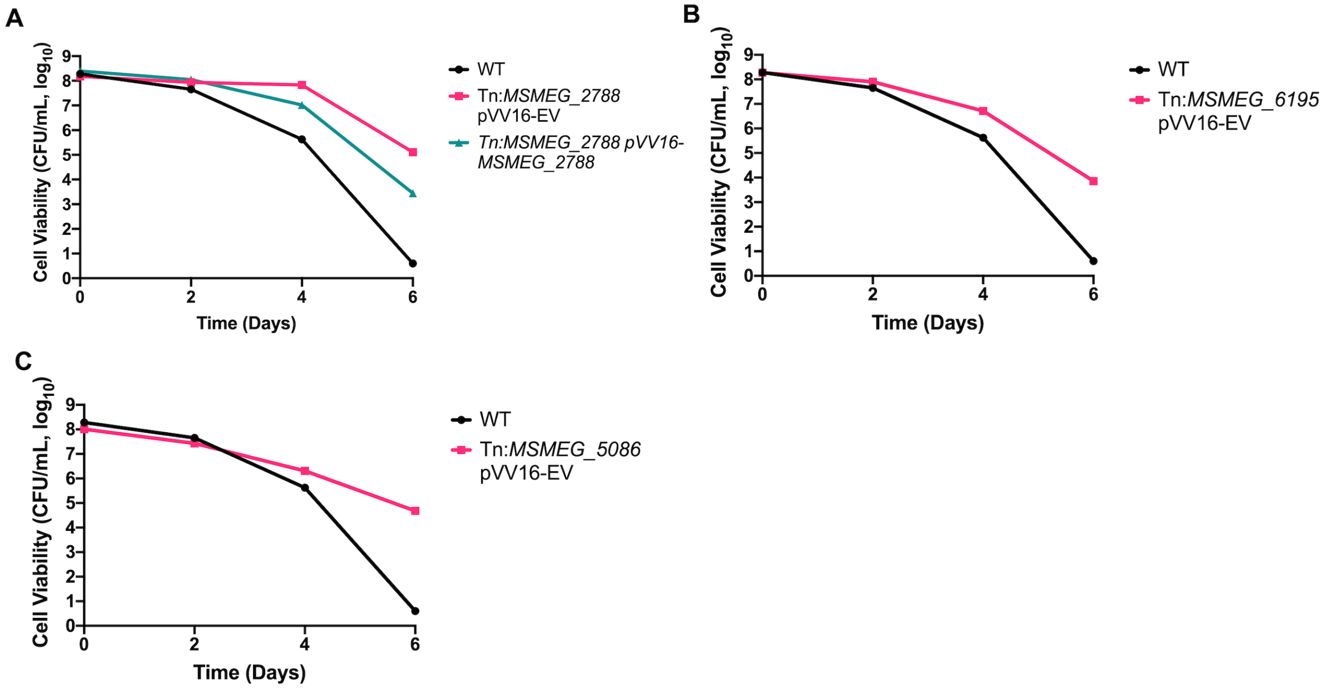

Supplemental Figure 4

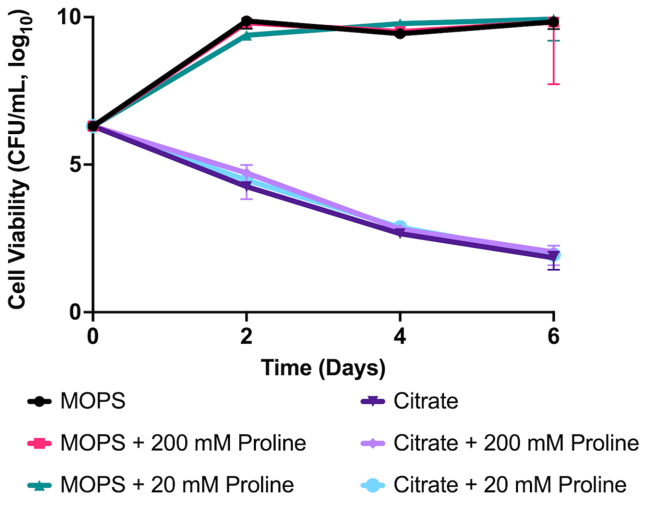

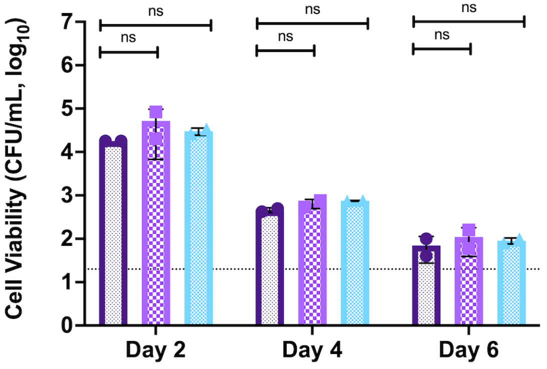

Supplemental Figure 5
